# The effect of bone cement distribution on clinical efficacy after percutaneous kyphoplasty for osteoporotic vertebral compression fractures

**DOI:** 10.1097/MD.0000000000018217

**Published:** 2019-12-16

**Authors:** Shuangjun He, Yijian Zhang, Nanning Lv, Shujin Wang, Yaowei Wang, Shuhua Wu, Fan He, Angela Chen, Zhonglai Qian, Jianhong Chen

**Affiliations:** a Department of Orthopedics, The People's Hospital of Danyang, Danyang; b Department of Orthopedics, The First Affiliated Hospital of Soochow University, Suzhou, China; c University of Waterloo, Waterloo, Canada.

**Keywords:** distribution of bone cement, osteoporotic vertebral compression fracture, percutaneous kyphoplasty

## Abstract

To evaluate the influence of various distributions of bone cement on the clinical efficacy of percutaneous kyphoplasty (PKP) in treating osteoporotic vertebrae compression fractures.

A total of 201 OVCF patients (30 males and 171 females) who received PKP treatment in our hospital were enrolled in this study. According to the characteristic of cement distribution, patients were divided into 2 groups: group A (“H” shaped group), the filling pattern in vertebral body were 2 briquettes and connected with / without cement bridge; and group B (“O” shaped group), the filling pattern in vertebral body was a complete crumb and without any separation. Bone mineral density, volume of injected cement, radiographic parameters, and VAS scores were recorded and analyzed between the 2 groups.

All patients finished at least a 1-year follow-up and both groups had significant improvement in radiographic parameters and clinical results. No significant differences in BMD, operation time, bleeding volume, or leakage of cement were observed between the 2 groups. Compared with group B, group A had a larger use of bone cement, lower proportion of unipedicular approach, and better VAS scores at 1 year after surgery.

Both “H” and “O” shaped distribution pattern can improve radiographic data and clinical outcomes effectively. However, “H” shaped distribution can achieve better clinical recovery at short-term follow-up.

## 1 Introduction

Osteoporotic vertebral compression fracture (OVCF) is a common disorder among elder people, which is regarded as one of most severe complications of osteoporosis. Menopause and low-trauma fracture are considered to be the major risk factors of low bone mineral density and fractures caused by bone frailty.^[1]^ The latest studies demonstrated that higher dietary glycemic index (DGI) and dietary glycemic load (DGL) can also increase the incidence of OVCF in elderly populations.^[2]^ It is noteworthy that the risk of recurrent fractures after the previous 1 can increase by 4 times, and almost one-third of patients will sustain another fracture within 5 years.^[3]^ Although conservative treatments such as oral analgesics and bed rest can alleviate acute pain, surgical treatment is still the optimal choice for most OVCF patients, especially for those with a deficit of neural function.^[4,5]^

As an improved technology from percutaneous vertebroplasty, percutaneous kyphoplasty has been recommended as an advanced procedure for treating OVCF in recent decades.^[6]^ Compared with PVP, PKP is proved to have a better correction of height of injured vertebrae and local kyphotic angle, without increasing the rate of leakage of bone cement and adjacent segment degeneration.^[7]^ However, previous literature has revealed that several factors may affect the outcomes after PKP treatment. Lin et al found a correlation between bone cement fraction and clinical outcomes and proposed the use of a quarter of the volume in unilateral kyphoplasty.^[8]^ Jeong et al indicated that insufficient penetration of bone cement into the trabecular bone may increase the risk of refracture of the cemented vertebrae.^[9]^ Meanwhile, some studies revealed that different distribution of bone cement may be related with better vertebral restoration and clinical outcomes. Lin et al reported that bone cement diffused over the contralateral pedicle could lead to best reconstructive effects.^[10]^ Yu et al pointed a comparatively diffused pattern may show better long-term outcomes for OVCFs with intervertebral cleft.^[11]^ Unfortunately, the optimal pattern of cement diffusion is not elucidated.

In this retrospective study, we compared many perioperative parameters among 2 different distribution of bone cement (“H” shaped and “O” shaped group) in PKP treatment to evaluate the influence of bone cement distribution on clinical efficacy.

## 2 Material and methods

### 2.1 Ethical statement

The present study was approved by the ethical committee of the First Affiliated Hospital of Soochow University and in accordance with Helsinki Declaration. Meanwhile, written informed consent was obtained from all the enrolled patients.

### 2.2 Patients selection

**Figure 1:**
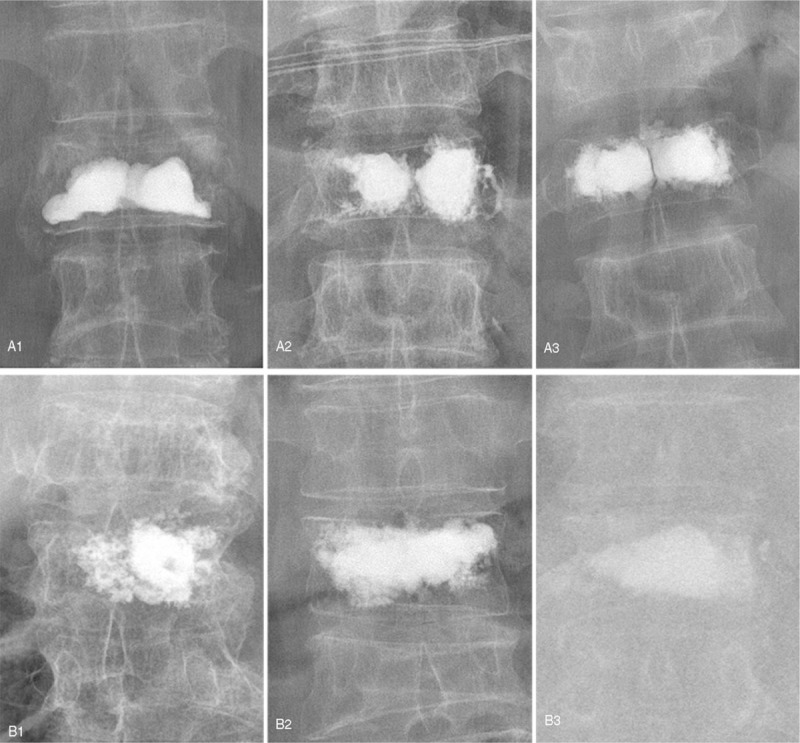
“H” shaped distribution of injected cement (a1, a2, a3): the bone cement in the vertebral body presented 2 briquettes, connected with or without a small amount of bone cement. “O” shaped distribution of bone cement (b1, b2, b3): the bone cement in the vertebral body presented a whole crumb, no separation or loss of bone cement.

In this study, we enrolled 201 patients who diagnosed with osteoporotic vertebral compression fracture (30 males and 171 females, mean age 69.1 ± 8.9) retrospectively between January 2015 and June 2017. All patients were treated with PKP and were placed into 2 groups according to the different distribution pattern: group A (“H” shaped group, 92 females and 18 males), the filling pattern in vertebral body were 2 briquettes and connected with/without cement bridge; and group B (“O” shaped group, 79 females and 12 males), the filling pattern in vertebral body was a complete crumb and without any separation (as shown in Fig. 1). Patients who met following criteria were enrolled:

1. definitive diagnosis of OVCFs on X-ray or CT and patients suffered from back pain;
2. single segmental OVCF at the thoracic or lumbar spine;
3. bone mineral density less than – 2.0;
4. without history of PKP surgery;
5. at least 1 year of follow-up.

The exclusion criteria were as follows:

1. damage of spinal cord or nerve root, with or without a neural disorder;
2. spinal metastatic tumor;
3. hemorrhage diseases like hemophilia;
4. other severe or systematic diseases;
5. insufficient data or loss to follow-up.

### 2.3 Surgical procedure

All patients received the PKP procedure by unilateral or bilateral approach for OVCF under general anaesthesia. The C-arm was applied for capturing anterior / posterior and lateral images for the surgical vertebrae. The bilateral or unilateral puncture trajectory were located at the posterior wall of the vertebrae. After that, the guide wires, expansion pipe, and working channel were sequentially inserted and inflatable balloons were placed into the vertebrae. Following, the prepared polymethylmethacrylate cement was slowly inserted into the vertebral body through a cannula with the perspective monitoring. This injection was stopped when filling cement was approached to the anterior wall of the vertebrae, and the cannula was then removed. All patients were allowed to walk 12 hours after surgery.

### 2.4 Evaluation method

Age, gender, BMD (T value), distribution of surgical segments, and surgical approach were recorded from each patient. Surgical details including the volume of injected bone cement, operative duration, blood loss, and occurrence of cement leakage were recorded. The radiographic parameters including the anterior vertebral height (AVH), anterior vertebral height ratio (AVHR), middle vertebral height (MVH), middle vertebral height ratio (MVHR), the local kyphosis angle (Cobb angle) were measured and analyzed at preoperative, 2 days after surgery, and 1 year after surgery. Clinical outcomes were assessed using VAS scores at preoperative, 2 days after surgery, and 1 year after surgery.

### 2.5 Statistical analysis

SPSS 17.0 software (SPSS Inc, USA) was used to analyze the data in our study and the results were presented as the mean and SD. Chi-Squared test was adapted to analyze the categorical variables, while Student *t* test was used to analyzes the continuous variables. Significant differences were defined as a *P* value less than.05.

## 3 Results

### 3.1 Demographic data

All patients received PKP treatment for single level OVCF and finished mean 19.1 months of follow-up (range from 12 to 28 months). There was no significant difference in age, sex, BMD between group A and group B. The surgical approach and surgical levels differed significantly between 2 groups (*P* < .001) (as shown in Table 1).

**Table 1:**
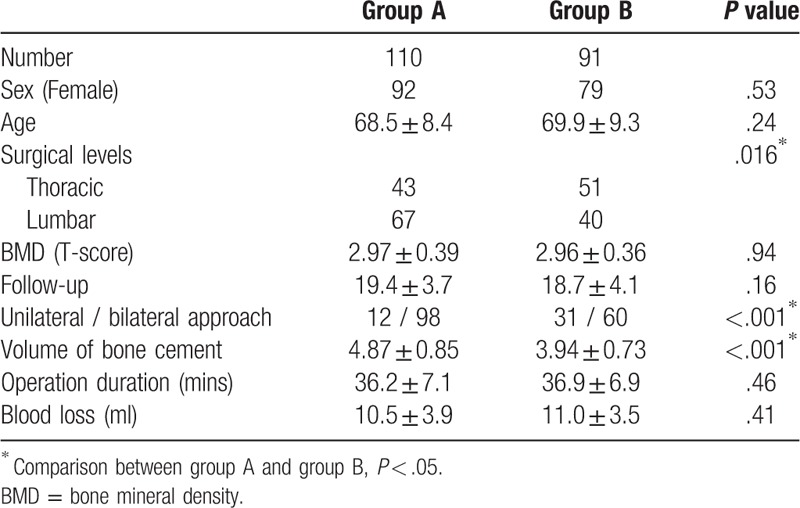
Demographic data.

### 3.2 Surgical details

No significant difference in operation duration and blood loss were detected between group A and group B. However, the volume of injected bone cement in group A was higher than in group B with significant difference (*P* < .001) (as shown in Table 1).

### 3.3 Radiographic data

**Figure 2:**
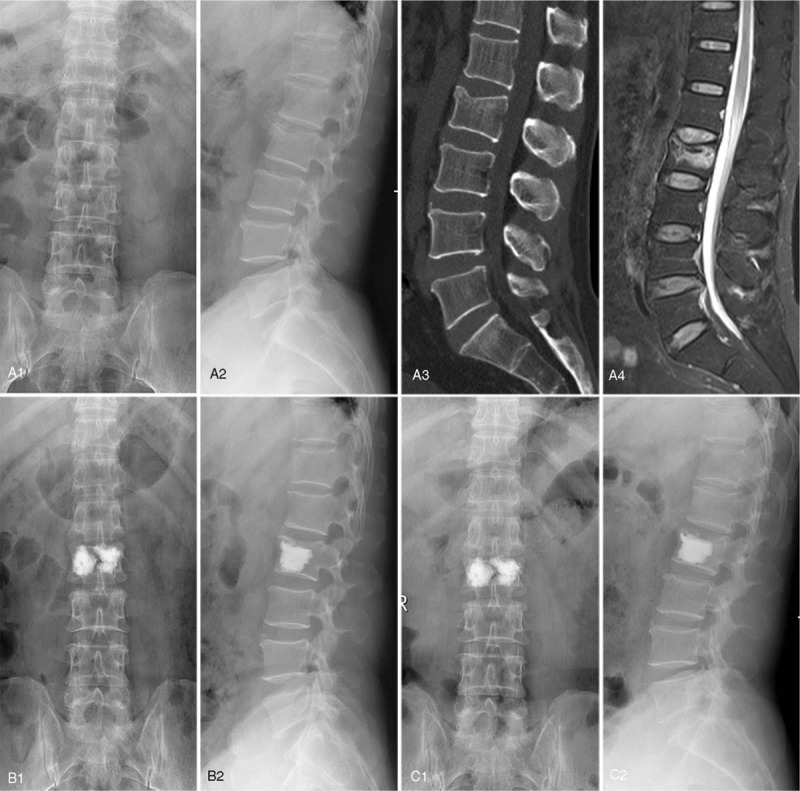
A 57-year-old male diagnosed as L2 OVCF with “H” shaped cement filling pattern. Preoperative X-ray from coronal plane (a1) and sagittal plane (a2), CT image (a3), and MRI fat suppression image (a4). Postoperative X-ray from coronal plane (b1) and sagittal plane (b2) at 2-days follow-up. Postoperative X-ray from coronal plane (c1) and sagittal plane (c2) at 1-year follow-up.

**Figure 3:**
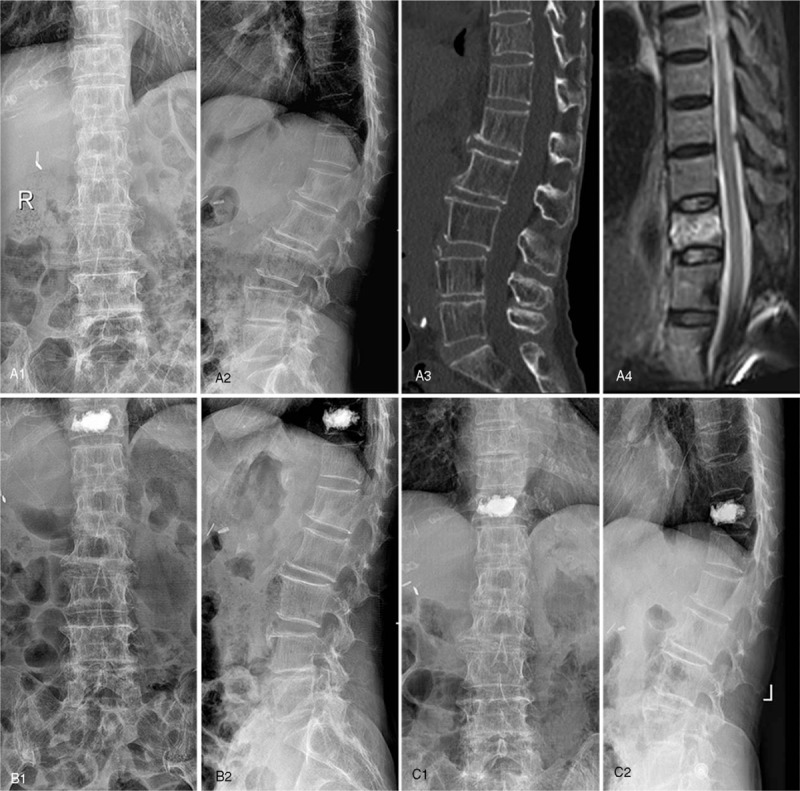
A 77-year-old female diagnosed as T11 OVCF with “O” shaped cement filling pattern. Preoperative X-ray from coronal plane (a1) and sagittal plane (a2), CT image (a3), and MRI fat suppression image (a4). Postoperative X-ray from coronal plane (b1) and sagittal plane (b2) at 2-days follow-up. Postoperative X-ray from coronal plane (c1) and sagittal plane (c2) at 1-year follow-up.

AVH, AVHR, MVH, and MVHR were all significantly restored at 2 days and 1 year after surgery, compared with the preoperative data in both groups (*P* < .001). Similarly, Cobb angle improved significantly after surgery for both groups (*P* < .001). There existed no significant difference in these radiographic parameters between 2 days and 1 year after surgery (as shown in Figs. 2 and 3).

Comparing within 2 groups, no significant difference was found in AVH, AVHR, MVH, and MVHR at preoperative, 2 days, and 1 years after surgery. Likewise, the Cobb angle did not differ significantly at the preoperative and each follow-up period (as shown in Table 2).

**Table 2:**
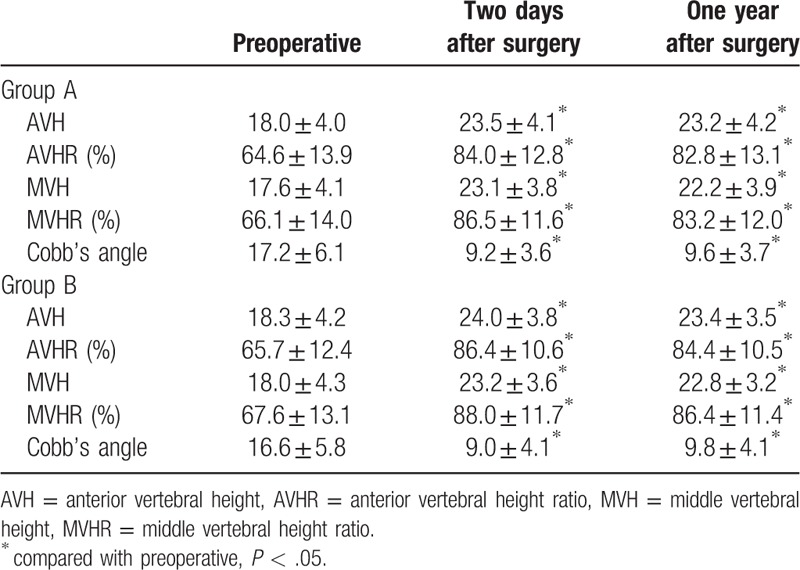
Radiographic parameters.

### 3.4 Clinical outcomes

Group A and group B both had significant improvement in VAS scores at 2 days and 1 year after surgery compared with preoperative in intra-group comparisons. VAS scores did not differ significantly between 2 groups at preoperative and 2 days after surgery but differed significantly at 1 year after surgery in inter-group comparisons (*P* < .001). Eight cases (8/110, 7.3%) in group A occurred intraoperative leakage of bone cement (3 cases with intradiscal leakage, and 5 cases with paravertebral leakage). Six cases (6/91, 6.6%) in group B occurred intraoperative leakage of bone cement (3 cases with intradiscal leakage, and 3 cases with paravertebral leakage). No case had spinal canal leakage or symptomatic leakage. There was no statistical difference in the proportion of leakage of bone cement between the 2 groups (as shown in Table 3).

**Table 3:**
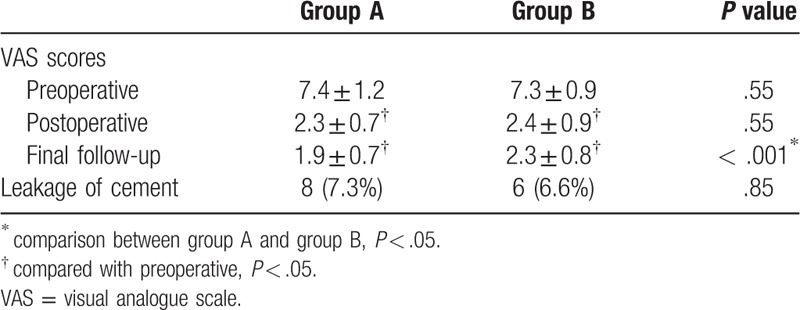
Clinical outcomes.

## 4 Discussion

Due to the improvement of balloon inflation, PKP can create a larger intervertebral space, better restoration of kyphotic angle, and lower incidence of bone cement extravasation.^[12]^ Previous researchers have indicated that during the PKP procedure, the volume of injected bone cement is markedly associated with the surgical efficacy.^[13]^ However, few studies reported on the impact of bone cement distribution on radiographic and functional recovery after PKP treatment. Yu et al reported that solid lump cement distribution pattern can cause a 12.5-fold higher risk of re-collapsed augmented vertebrae after PVP treatment for OVCF with intervertebral cleft.^[11]^ He et al also verified that the higher incidence of recompression in the cemented vertebrae can be detected in bone cement distribution of uninterlocked solid pattern and discontiguous trabecular pattern after PVP.^[14]^ In present study, an obvious bigger proportion of females were enrolled, indicating females were more likely to suffered from OVCFs owing to menopause. We found that several parameters showed a predominant improvement after PKP treatment in both groups (radiographic data and clinical outcomes), indicating that PKP procedure can effectively restore the sagittal balance and relieve painful symptoms. No significant difference was shown in Cobb angle, elative height of the anterior margin of the injured vertebra, and the relative height of the middle of the injured vertebrae between 2 groups, which means that 2 types of distribution can achieve concordant reconstruction of the biomechanical stability of spine. Notably, group A achieved significantly greater VAS scores than group B at the 1-year follow-up, which suggests that the “H” shaped distribution is accompanied with better mid-term analgesic effects. We speculated that “H” shaped distribution of bone cement has a wider contact area with cancellous bone in augmented vertebrae, which can reduce the adverse effect of chemical heat release on mating surface and increase the “binding” function between bone cement and trabecula bone.^[15]^ Moreover, the loading pattern in “H” shaped distribution is close to plateau support rather than the pitting support in “O” shaped distribution, which can increase the stability of vertebrae, decrease the micro motion of the trabecular bone, and relieve the remaining back pain.^[16]^

A biomechanical study focused on the impact of volume on vertebral stiffness in PVP and found that approximately 16% cement volume filling can restore vertebral strength, while approximately 29% cement volume filling can restore vertebral stiffness.^[17]^ Other than the volume of injected cement, the distribution or location of the cement may also affect the strength and stiffness of the spine:

1. **Symmetry:** An experimental study by Chen et al found that cement augmentation can be limited to only 1 side of vertebral body in unipedicular PKP, thus the stiffness in the non-augmented side may be reduced at various degrees.
2. **Balance:** The imbalanced distribution can subsequently result in imbalanced mechanical load in augmented vertebrae and bring several detrimental alterations;^[18]^ for example, abnormal load transfer within injured vertebrae will cause the accelerated rate of failure of the adjacent segment.^[19]^
3. **Load:** In comparison with “O” shaped distribution, the touching area in “H” shaped distribution is dramatically bigger on the endplate. Hence, under the same loading pressure, the bigger contact area in latter one can reduce the damage in surrounding bone through the prevented damage accumulation in filled regions and hampered collapse of vertebrae.^[20]^
4. **Interaction:** The “H” shaped distribution can achieve more sufficient interactions between cancellous bone and cement and provide better vertical support that can decrease the incidence of recompression after surgery.^[21]^ Likewise, the more adequate cement filling in “H” shaped distribution can lead to superior fixation of fracture fragments that relieve pain better.^[22]^

The different distribution of bone cement in PKP is multifactorial, and we inferred that it may be attributed to the following reasons: first, different approaches may result in different cement morphologies. In this study, there was a significant difference in surgical approach between the 2 types of distribution. The proportion of unipedicular approach in group B was dominantly higher than in group A, indicating that the former predisposes to generate isolated cement lump that cause the asymmetrical distribution of bone cement. Second, different distribution of bone cement may be related to volume and properties of cement injection. Our study showed a bigger volume of cement within group A, which we speculated to result from 2 factors: the first is the higher proportion of unipedicular patients in group B, as mentioned above. The other factor is that the surgical segments in group B were intended to be distributed at a high-level of the spine with less vertebral cavity (thoracic spine), which may lead to less use of cement in group B. As for the properties, it was considered that high-viscosity cement can achieve a better spread in the body of vertebrae more homogeneously and reduce of risk of leakage compared with low-viscosity.^[23]^ Third, the puncture point is also a key factor. It is reported that in unilateral PKP, a more lateral facet joint puncture point with larger extraversion angle can form a distribution of bone cement in anterior and middle area in the vertebral body. Moreover, this distribution can better restore the kyphotic angle of injured vertebra.^[24]^ Ultimately, the operating time may affect the distribution of bone cement. Previous studies indicated that pre-existing defects of the fracture gaps can be filled with soft tissues, including organized hematoma and fibrous scars at 2 weeks after the fracture, while proliferating tissues can hinder the homogeneous diffuse of bone cement.^[25]^

The present study still has several limitations that should be considered. First, our study was retrospective with a small sample size and short-term follow-up. Second, patients were grouped according to their X-ray instead of CT due to insufficient data, which may have led to some assessed errors. Third, no previous studies reported the same grouping method as ours, which may cause subjective bias in the categorizing process. Fourth, the discrepancy of injected bone cement may introduce to some bias between groups. Hence, larger samples and multiple center studies which focus on the impact of cement distribution in PKP are required in the future.

## 5 Conclusion

This study preliminarily confirmed that both “H” shaped distribution and “O” shaped distribution of bone cement can achieve satisfied improvement in radiographic and clinical outcomes after PKP.“H” shaped can obtain a better clinical recovery in mid-term follow-up than “O” shaped distribution.

## Author contributions

**Data curation:** Shuangjun He, Yijian Zhang.

**Formal analysis:** Nanning Lv.

**Methodology:** Shujin Wang.

**Resources:** Yaowei Wang.

**Software:** Shuhua Wu.

**Supervision:** Fan He.

**Validation:** Angela Chen.

**Writing – review & editing:** Zhonglai Qian, Jianhong Chen.
